# Fabrication and Qualitative Analysis of an Optical Fibre EFPI-Based Temperature Sensor

**DOI:** 10.3390/s21134445

**Published:** 2021-06-29

**Authors:** Fintan McGuinness, Aidan Cloonan, Mohamed Oubaha, Dinesh Babu Duraibabu, M. Mahmood Ali, Gerald Kilkelly, Emma Tobin, Gabriel Leen

**Affiliations:** 1 Department of Electronic and Computer Engineering (ECE), University of Limerick, V94 T9PX Limerick, Ireland; Mahmood.Ali@ul.ie (M.M.A.); Emma.Tobin@UL.ie (E.T.); Gabriel.leen@ul.ie (G.L.); 2 Bernal Institute, University of Limerick, V94 T9PX Limerick, Ireland; aidan.cloonan@ul.ie (A.C.); gerald.kilkelly@ul.ie (G.K.); 3 Centre for Research in Engineering Surface Technology (CREST), Technological University Dublin, D08 CKP1 Dublin, Ireland; mohamed.oubaha@tudublin.ie; 4 Centre for Robotics and Intelligent Systems (CRIS), University of Limerick, V94 T9PX Limerick, Ireland; dineshbabu.duraibabu@ul.ie

**Keywords:** optical, fibre, EFPI, temperature, sensor, polymer, high sensitivity

## Abstract

The following presents a comparison of an extrinsic Fabry–Perot interferometer (EFPI)-based temperature sensor, constructed using a novel diaphragm manufacturing technique, with a reference all-glass EFPI temperature sensor. The novel diaphragm was manufactured using polyvinyl alcohol (PVA). The novel sensor fabrication involved fusing a single-mode fibre (SMF) to a length of fused quartz capillary, which has an inner diameter of 132 μm and a 220 μm outer diameter. The capillary was subsequently polished until the distal face of the capillary extended approximately 60 μm beyond that of the single mode fibre. Upon completion of polishing, the assembly is immersed in a solution of PVA. Controlled extraction resulted in creation of a thin diaphragm while simultaneously applying a protective coating to the fusion point of the SMF and capillary. The EFPI sensor is subsequently sealed in a second fluid-filled capillary, thereby creating a novel temperature sensor structure. Both temperature sensors were placed in a thermogravimetric analyser and heated from an indicated 30 °C to 100 °C to qualitatively compare sensitivities. Initial results indicated that the novel manufacturing technique both expedited production and produces a more sensitive sensor when compared to an all-glass construction.

## 1. Introduction

In biomedical applications, the use of optical fibre sensors has become increasingly prevalent in the past decade, with significant industry players interfacing with universities to further develop such sensors [1]. Driving factors for this include the small dimensions of optical fibre sensors, their immunity to electromagnetic interference, and general ease of achieving biocompatibility [2,3]. The aforementioned benefits are of particular advantage in radiotherapy linear accelerators [4,5,6], magnetic resonance imaging (MRI) devices [7,8,9], and general in vivo applications themselves [10,11,12]. In addition to the aforementioned patient-centric applications, high-resolution temperature sensors may also be of benefit in micro-volume calorimetry applications. Their small form factor makes them minimally invasive, and the glass-based outer construction is easily sterilised, which helps minimise the risk of contamination. The temperature resolution of the optical fibre sensors are also comparable to that of quartz thermometers (1 × 10^−3^–1 × 10^−5^ K) [13,14].

**Figure 1:**
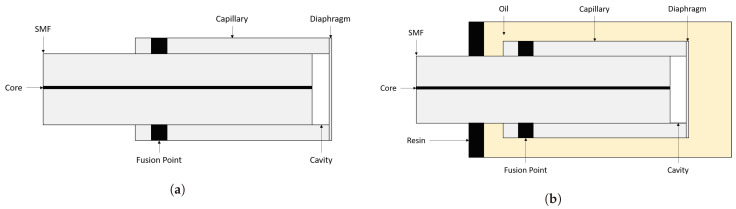
(**a**) Schematic of OFPS sensor based on McGuinness et al. (Adapted from ref. [16]). (**b**) Schematic of UHRTS sensor based on Poeggel et al. (Adapted from ref. [15]).

Previous research at the Optical Fibre Sensors Research Centre, University of Limerick, has resulted in numerous publications and several patents relating to the design, manufacture, and application of optical fibre pressure and temperature sensors. Efforts have been made to improve upon the relatively complicated manufacturing process of the sensor’s silica diaphragm, and much research effort has been expended in the finding of alternative materials and methods of production. A schematic of the current all-glass design is presented in Figure 1. The manufacturing of silica diaphragms has limited the scalability of production, which in turn has limited research opportunities owing to a significant manufacturing lead time. Here, we report on work undertaken on one method to reduce the time required to manufacture an Extrinsic Fabry-Perot Interferometer (EFPI), which may potentially be used in an Ultra-High-Resolution Temperature Sensor (UHRTS) similar to that of Poeggel et al. [15], while simultaneously improving its mechanical resistance to failure modes resulting from eternally applied shear stress to the sensor. A further objective of the work reported here is the removal of the requirement to etch a silica diaphragm in hydrofluoric acid (HF) in order to achieve optimal diaphragm thickness, as HF is a hazardous substance.

**Figure 2:**
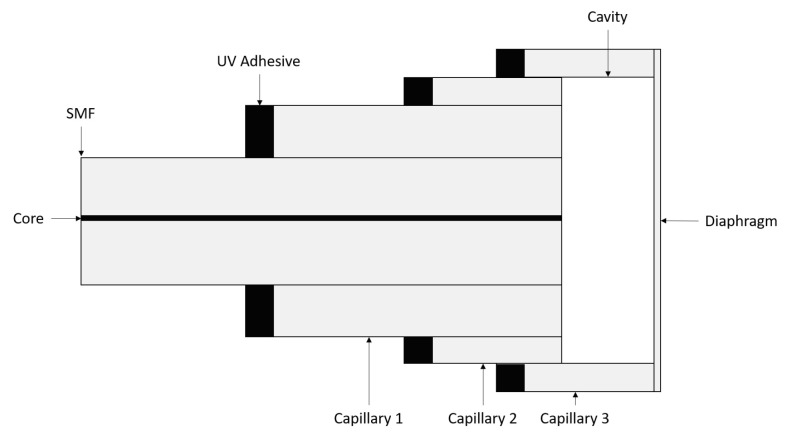
Schematic of an EFPI sensor based on Li et al. (Adapted from ref. [17]).

Several methods have been reported in the literature for creating high-sensitivity diaphragms on EFPIs. These have typically been an involved process requiring several sub-assemblies relying on adhesives, micro-machining, or multiple fusion steps [17,18,19,20]. One such EFPI is that proposed by Li et al. [17], where the diaphragm thickness is in the region of 8 μm. To produce the sensor of Li et al., an SMF is bonded within three concentric glass tubes using a UV curable adhesive until the sub-assembly has an outer diameter of 10 mm. The sub-assembly forms the proximal end of an EFPI and an EFPI cavity, which is open at the distal end. The distal end of the EFPI is created by applying adhesive to the end face of the outermost glass tube and placing a prefabricated polyethylene diaphragm on the sensor. While this technique presented by Li et al. removes the requirement of HF etching and offers a good diaphragm aspect ratio, there is still significant fabrication in the production and alignment of the precise tubing used. A schematic of the sensor is provided in Figure 2.

**Figure 3:**
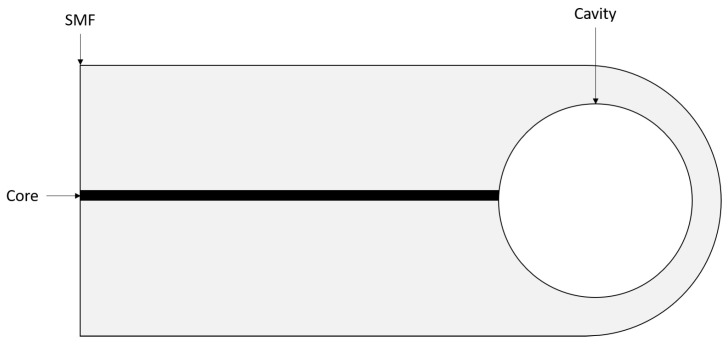
Schematic of an EFPI sensor based on Liao et al. (Adapted from ref. [18]).

Liao et al. addressed the issue of complex assemblies by forming a sub-micron diaphragm at the end of two single-mode fibres (SMF) simultaneously using only a fusion splicer and index matching gel [18]. Initially, two SMFs are exposed to an arc in the fusion splicer to create hemispherical end faces, before being coated in an index matching gel. After coating in the index matching gel, the SMFs are aligned in the fusion splicer and an arc applied to the SMFs, while the hemispherical end faces are pushed together to form a large central bubble in the fused fibres. A further arc is then applied to the large bubble as the fibres are withdrawn from each other until the large central bubble collapses on itself, producing two smaller bubbles. The fibres are further withdrawn from each other until they separate, and a small bubble exists at the distal end of the two SMFs. A final arc is then applied to the small bubbles until they expand to the desired diameter. The resultant diaphragms are in the region of 500 nm thick, and these sensors exhibit a low temperature cross-sensitivity between temperature and pressure. It is also claimed that for a given diaphragm thickness, the spherical-like structure results in greater mechanical strength when compared to traditional EFPI manufacturing techniques. Furthermore, the sensor construction presented by Liao et al. has the particular advantage of being significantly smaller than most traditional EFPI sensors as it is fabricated entirely from a single SMF (two fibres are required, but it results in two sensors). Another advantage of this construction is that the time exposed to the final fusion arc controls the cavity bubble size and by extension the diaphragm thickness. However, due to the manufacturing method, the spherical diaphragm itself is not of uniform thickness, which can result in a non-linear sensor response. A schematic of the final sensor head is presented in Figure 3.

**Figure 4:**
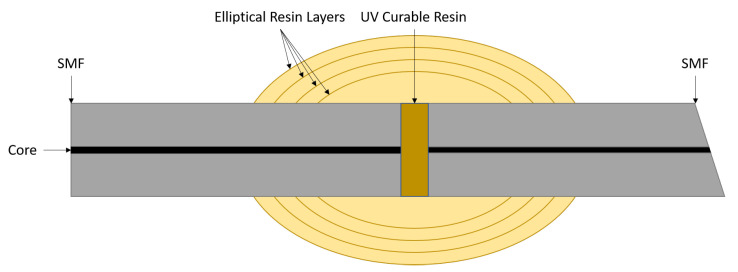
Schematic of an EFPI hydrostatic pressure sensor based on Oliveira et al. (Adapted with permission from ref. [19]. Copyright 2019 IEEE).

Similar to the concern regarding diaphragm shape noted by Liao et al. [18], Oliveira et al. report a method to ensure symmetry in their elliptical, polymer pressure sensor [19]. The embodiment of the sensor presented by Oliveira et al. exists somewhere between that of Bremer et al. [21] and Li et al. [17], where the proximal and distal mirrors are made of silica. However, in the case of Oliveira et al., the EFPI cavity is filled with a flexible UV curable resin; i.e., the aligned SMFs are bonded using the resin. Following the bonding of the SMFs, the area surrounding the sensor cavity is then coated in successive elliptical layers of the same UV-curable resin. This resin has a two-fold effect on the behaviour of the sensor: (a) it strengthens the location where the two SMFs are bonded together; (b) the greater surface area of the elliptical resin results in a higher hydrostatic pressure sensitivity; i.e., a greater straining force is applied to the structure for a given pressure increase. A schematic of the sensor described by Oliveira et al. is presented in Figure 4.

**Figure 5:**
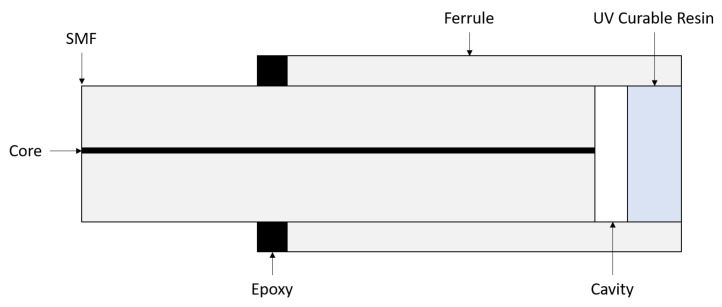
Schematic of EFPI sensor based on Zhang et al. (Adapted from ref. [20]).

Zhang et al. [20] removed the need for symmetry by developing an embedded diaphragm made from polydimethylsiloxane (PDMS) (see Figure 5). The embedded diaphragm is achieved by vertically submerging a diaphragm-less SMF-capillary assembly into a PDMS solution and removing it slowly, resulting in the formulation of a diaphragm approximately 9.6 μm thick. Although not as thin as the sensors developed by Liao et al. [18], the issue of a nonlinear response is removed, while still obtaining an adequate value for sensitivity. Similarly, the method of vertically submerging the diaphragm-less SMF-capillary assembly sensor head significantly reduces the previously mentioned manufacturing complexities. It is claimed that due to the fact the diaphragm is embedded, it has higher mechanical strength than a tip-based sensor. However, due to the viscous nature of PDMS and the capillary action of the hollow core fibre, the diaphragm continues to move toward the SMF until it is cured. This introduces a level of uncertainty regarding the cavity length, which is undesirable but is generally not an issue when dealing with tip based diaphragms.

Taking the aforementioned designs into consideration, the authors noted that there exists potential for a liquid based diaphragm manufacturing technique wherein there is no requirement for HF etching. In addition to the removal of HF etching, the cured liquid used may also act in such a way that it mechanically strengthens the other components of the final assembly. Finally, it is probable that the diaphragm would be planar in its construction and thus minimise potential non-linearity in its mechanical response, provided the diaphragm is not excessively deflected (where the deflection is approximately <10% of its thickness). Here, we discuss the use of a curable liquid as a means of forming a diaphragm on the end-face of a single mode fibre (SMF) and capillary assembly to create an optical fibre pressure sensor (OFPS). Subsequently, the pressure sensor created is used as a sub-component of a novel temperature sensor. The material selected to form the diaphragm is polyvinyl alcohol (PVA). An SMF-capillary assembly is immersed to a sufficient depth such that the PVA coats the fusion point of the assemblies in addition to forming a diaphragm. By doing so, the PVA creates a protective layer at the stress concentration point (SMF-capillary fusion point) in addition to creating the diaphragm. After successful creation of the diaphragm, the OFPS sensor is sealed in a second oil filled outer capillary to create a temperature sensor. The temperature sensor’s performance is compared to that of a reference all-glass UHRTS manufactured in a similar manner to that reported by Poeggel et al. [15], where the diaphragm is glass. The sensor is interrogated between 1510 and 1590 nm, and a qualitative comparison of the temperature response is performed by monitoring the spectral response as a function of increasing the temperature in a Perkin Elmer TGA 4000 thermogravimetric analyser (TGA). The monitoring of the spectral response is achieved through tracking the reflection intensity at an arbitrary wavelength on a linear portion of a sensor’s baseline EFPI interference spectrum.

This paper is structured as follows; Section 2 presents the materials and methods used to manufacture and test the two temperature sensors. The methods discussed in Section 2 include manufacturing of the sensor diaphragm-less SMF-capillary assembly, creation of the novel diaphragm, assembly of the temperature sensor, and testing of the temperature sensor in a TGA. Section 3 presents the results recorded during the TGA-based analysis of the sensors. Section 4 discusses the results presented in Section 3 and the potential merits of using the novel diaphragm versus the reference all-glass construction.

## 2. Materials and Methods

The following section is divided into five subsections, the first three of which describe preparation of the optical fibre components used to create the sensors. The remaining two subsections discuss the interrogation and experimentation carried out with the sensors. While an all-glass sensor was used as a reference for this study, the manufacturing process is not presented here as it was not the focus of this discussion and can be found in Bremer et al. [21,22].

### 2.1. Preparation of Optical Components

A length of fused quartz capillary with internal and outer diameters of 132 μm and 220 μm respectively, is trimmed using a motorised diamond blade cleaver to approximately 2 mm in length. The capillary is then placed into one end of a fusion splicer with the trimmed portion of the capillary facing the fusion electrodes. Care is taken to minimise fractures at the trimmed end as these fractures may cause damage to the sensor during the early phases of manufacturing. The next step involves preparing a length of single mode fibre (SMF 28e+) such that a portion of its protective jacket is removed and is cleaved to approximately 8 mm in length using a field cleaver. The stripped portion of SMF is then placed into the opposing end of the fusion splicer and is coaxially aligned with the capillary. The aligned SMF is then introduced into the capillary until there is sufficient overlap to successfully fusion splice the SMF to the capillary. The splicer used in this study was an Ericsson (Kista, Sweden) FSU 975, and the settings used to fuse the SMF and capillary were provided in Table 1.

Upon removal from the fusion splicer, the SMF-capillary assembly was placed in a polishing ferrule. Significant care needs to be taken at this stage as the interface of the SMF and capillary is a mechanical stress concentration location, due to the mismatch in physical dimensions (component diameters). The mismatched diameters result in a dominant failure mode, which is shearing between the proximal end of the capillary and distal end of the SMF. This is mitigated in a later step by coating and mechanical reinforcement. Once successfully inserted into the polishing ferrule, the capillary is scribed using a sapphire blade such that the new distal end-face of the capillary is located approximately 100–200 μm from the end-face of the SMF. At this point, the SMF is connected to an optical spectrum analyser (OSA), while the end face of the capillary is polished. The exposure period of the Ibsen (Farum, Denmark) I-MON 512 OSA was set to 1.5 ms, and the period between exposures was set to 50 ms. The light source used was an Exalos (Schlieren, Switzerland) EXS210069-01. The relatively large period between sensor integrations during polishing was acceptable due to the slow nature of the polishing process. As it takes approximately 30 min to reduce the capillary head from approximately 200 μm to the final length of 60 μm, there is no significant benefit to having a higher sample rate of the optical spectrum. The length of the cavity is confirmed by placing the polishing ferrule perpendicular to a planar glass plate. By doing so, an EFPI cavity is created ($L_{c}$) between the end-face of the SMF and surface of the glass plate. The length of the cavity may be determined using Equation (1), where $\lambda_{1}$ and $\lambda_{2}$ were the wavelengths of two neighbouring peaks in the generated EFPI spectrum.

(1) $$\begin{array}{c} L_{c}=\frac{\lambda_{1}*\lambda_{2}}{2(\lambda_{2}-\lambda_{1})} \end{array}$$

Reduction of the cavity length is carried out by polishing the end face of the capillary on increasingly fine grit polish films until the cavity is 60 μm in length. For a typical sensor, initial polishing is carried out on 12 μm grit film until the cavity is 150 μm in length; subsequently, 3 μm grit polish film is used to reduce the cavity length to approximately 100 μm. Finally, 1 μm grit polish paper is used to bring the cavity to a length of 60 μm. Once the polishing process is completed, the end face of the capillary is inspected to ensure that it is free from dust and damage. If dust is present in the capillary, then the assembly is placed vertically into a beaker filled with isopropyl alcohol (IPA), which is then placed in an ultrasonic bath. The capillary is sonicated for approximately 5 s to free debris, and it is then allowed to air-dry before being placed in a fusion splicer where an arc of 10 mA for 1 s is applied to remove any remaining debris or IPA trapped inside the capillary.

### 2.2. Novel Diaphragm Manufacturing Technique

**Figure 6:**
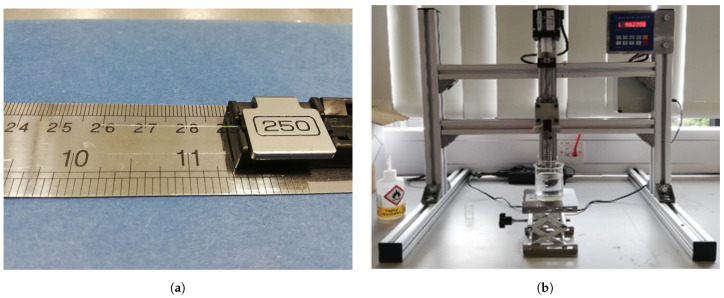
(**a**) Sensor blank extending approximately 30 mm from the fibre clamp, with 8 mm of exposed fibre. (**b**) Sensor immersion rig.

The diaphragm-less SMF-capillary assembly is placed into a 250 μm clamp, such as that commonly used in a cleaver or portable fusion splicer, with approximately 30 mm of the assembly extending beyond the clamp; see Figure 6a. It is not necessary to know the exact length of the overhang provided all of the exposed SMF is visible, this being approximately 10 mm of the overhang. Once the PVA is prepared, it is poured into a beaker until the liquid height is in excess of 40 mm. This allows for the fibre overhang to be inserted entirely into the fluid without risk of impacting the sensor head on the base of the beaker. The beaker is then placed on a vertical laboratory stage under a custom immersion rig; see Figure 6b below.

**Figure 7:**
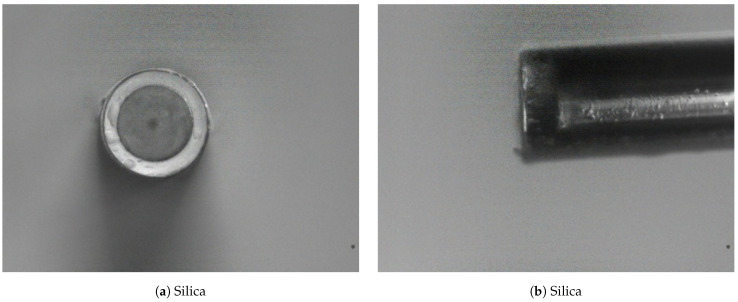

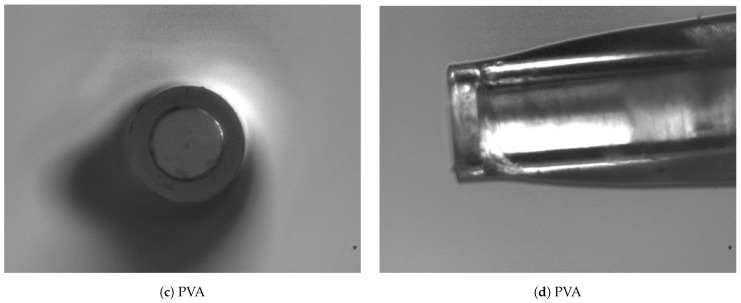
(**a**) Silica diaphragm axial view; (**b**) Silica diaphragm side view; (**c**) PVA diaphragm axial view; (**d**) PVA diaphragm side view.

Based on previous experiments and experience, the immersion rate was not found to be a significant factor in determining the quality of the diaphragm. Immersion time was set to 120 s based on the authors’ experience. The extraction rate was set to 40 mm/min as this produces a coating that is strong enough to protect the fusion location of the SMF and capillary, while simultaneously producing a diaphragm that is thin enough to produce a highly sensitive pressure sensor. In order to fully cure the PVA diaphragm, it is left at room temperature for 24 h. Figure 7 shows an axial and cross-sectional view of the sensor diaphragm manufactured using PVA, along with the reference all-glass sensor. When comparing the axial views, it is apparent that PVA diaphragms is of a similar transparency in the visible light range when compared to the all-glass reference sensor. Comparison of the cross-sectional views indicates that the silica sensor has a diaphragm in the region of 3 μm thick and the PVA diaphragm is in the region of 5 μm thick. Slight ingress of the PVA into the capillary resulted in a slightly dog-bowl-shaped concave diaphragm. While not observed to be an issue for reflectivity of the light beam exiting the SMF, the reduced diaphragm diameter would theoretically result in a stiffer diaphragm t, hereby reducing the potential diaphragm deflection z(r) as determined by Equation (2), where *z* is the diaphragm deflection, ν is the Poisson’s Ratio, *R* is the outer radius, *r* is the radius from the centreline, *E* is the Young’s Modulus, *h* is the diaphragm thickness and *P* is the stagnation pressure on the exterior face of the diaphragm. The PVA sensor, while having a thicker diaphragm compared to the reference all-glass sensor, also presented a thick tapered coating almost doubling the outer diameter of the sensor head at around 0.5 mm at its thickest along the capillary.

(2) $$\begin{array}{c} z\left(r\right)=\frac{3}{16}\;\cdot \;\frac{\left(1-\nu^{2}\right)\left(R^{2}-r^{2}\right)}{Eh^{3}}\;\cdot \;P \end{array}$$

### 2.3. Manufacturing the Temperature Sensor

**Figure 8:**
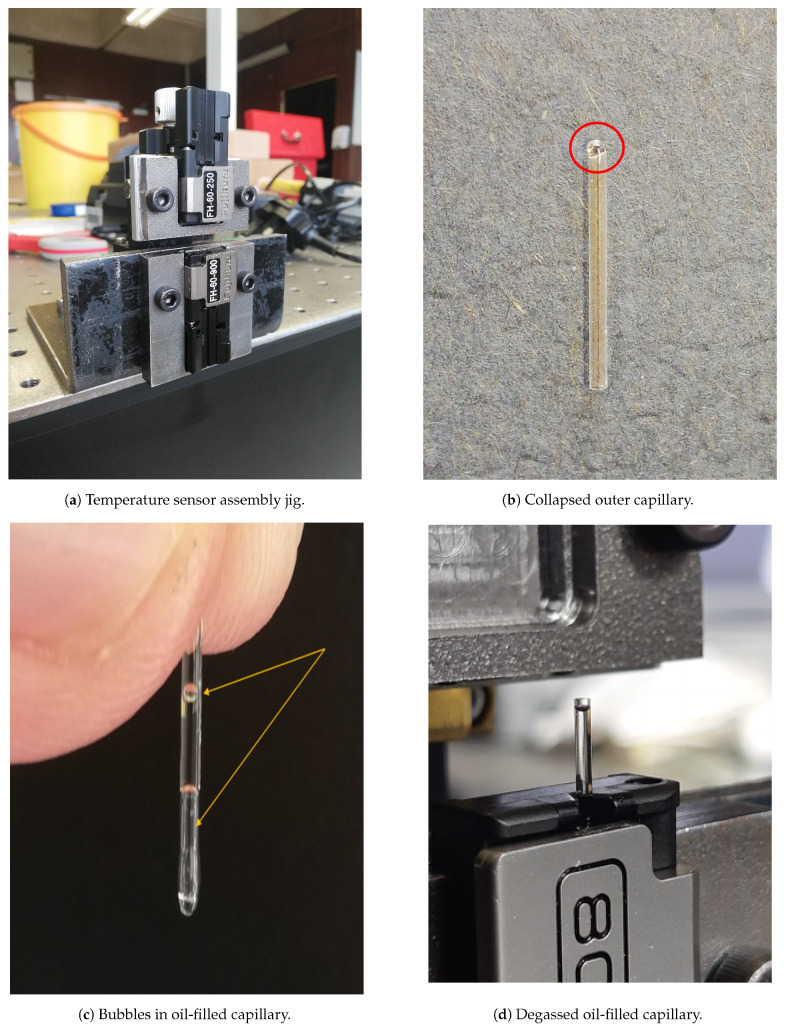
(**a**) Temperature sensor assembly rig; (**b**) Outer capillary with collapsed end face (circled); (**c**) Oil filled outer capillary with macro-bubbles present; (**d**) Degassed outer capillary.

Once the pressure sensor was deemed to be sufficiently cured, air dried for 24 h, and inspected under a microscope to ensure no major defects were present, it was placed into the upper fibre holder of the sensor manufacturing rig as illustrated in Figure 8a. Following this, preparation of the outer oil-containing capillary commenced. The fused quartz outer capillary has an internal diameter of 780 μm and an outer diameter of 1000 μm (1 mm). However, as the capillary is open ended, one end requires collapsing in order to ensure it is sealed at the distal end. This is done using a Siecor (Hickory, NC, USA) location. M90 fusion splicer using the settings presented in Table 2 below. In order to minimise the risk of a collapsed capillary bead falling into the splicer, the capillary was moved in and out of the arc intermittently to minimise hotspots. An image of a collapsed outer capillary is presented in Figure 8b.

The collapsed capillary is placed in the lower fibre clamp of the UHRTS manufacturing rig and approximately 2 mm below the OFPS; see Figure 8a. Following this, 5 W-40 SAE grade engine oil is injected into the outer capillary using a syringe and 25 Gauge blunt needle. Once the outer capillary is filled with oil, it is not uncommon to note an air bubble such as that shown in Figure 8c. While the bubble naturally dissipates over a period of hours, its removal may be expedited by introducing a flexible piano wire such as that produced by Fiber Instrument Sales (Oriskany, NY, USA). However, it is still recommended that the outer capillary be left as long as possible to de-gas potential micro-bubbles (e.g., 2 h). It is desirable to remove the air bubble as the air is more compressible than the oil. As the sensor is heated, the compression of air bubbles would serve to dampen the sensor’s response. Once the outer capillary is allowed to degas for a period of time (e.g., 2 h), the oil level is topped up as necessary and allowed to rest for a second period of time (e.g., 30 min). Figure 8d shows an outer capillary post macro-bubble removal and before oil top up.

**Figure 9:**
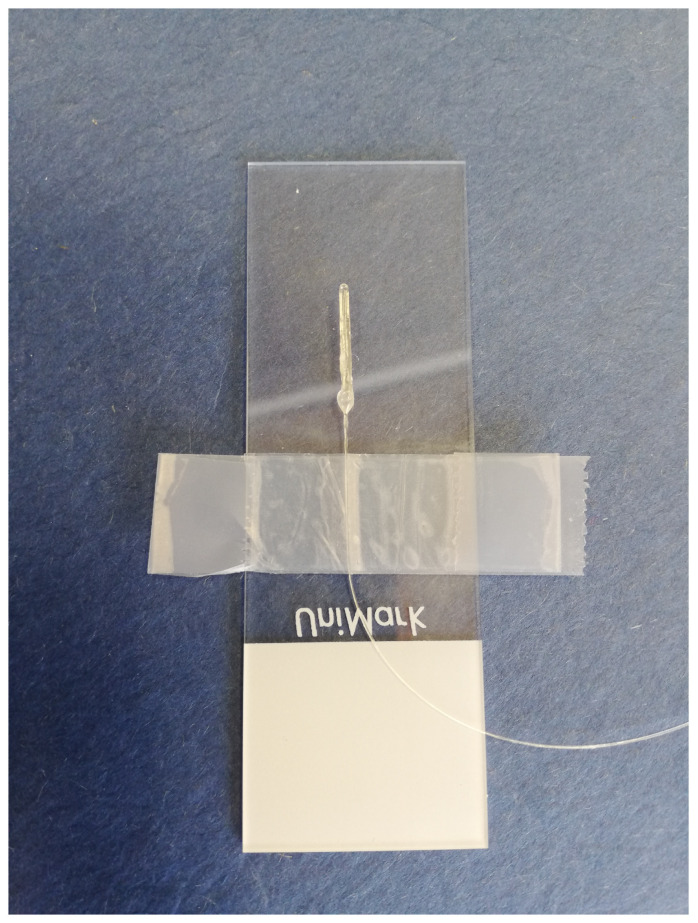
Finished UHRTS on a 75 × 26 mm microscope slide with SMF visible.

The pressure sensors are subsequently introduced into the outer capillaries using micro-stages, which the fibre clamps are connected to, such that the diaphragm is approximately at the centre of the outer capillary, both radially and longitudinally. Once the sensor is positioned in the desired location, the open end of the outer capillary is sealed using a two part epoxy adhesive. For the sensors presented herein, 3M (St. Paul, MN, USA) *Double Bubble* epoxy is used. A fabricated temperature sensor is illustrated in Figure 9.

### 2.4. Interrogation of the Temperature Sensors

**Figure 10:**
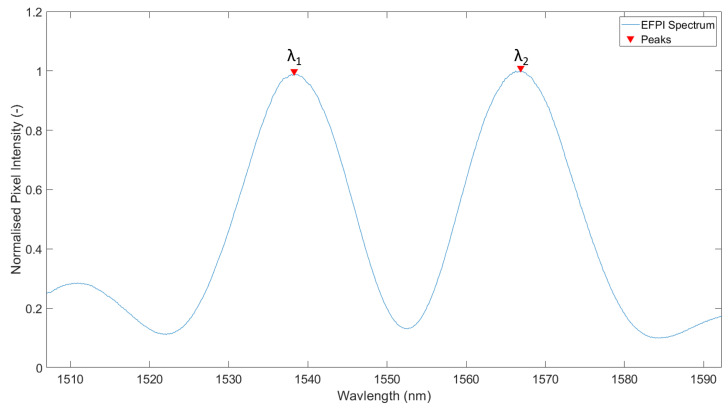
Sample EFPI interference pattern where peaks (signified by arrows) are approximately 30 nm apart, indicating a cavity length of 41 μm.

**Figure 11:**
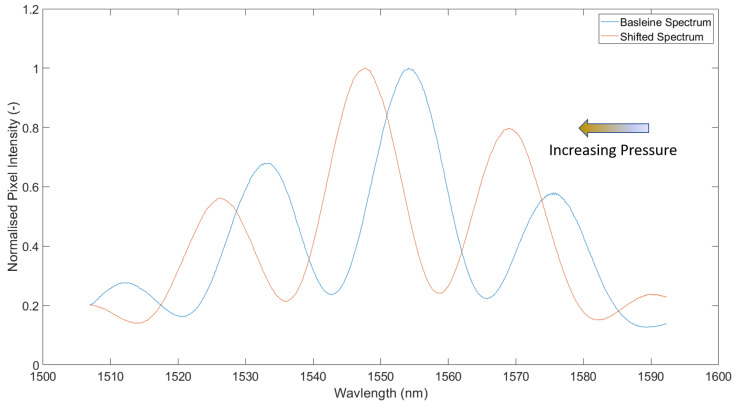
Example of EFPI interference pattern blue shifting as a result of decreasing cavity length due to increasing external pressure.

Both of the sensors are interrogated using a BaySpec (San Jose, CA, USA) WaveCapture FBG Interrogator. The range over which the sensors are interrogated is 1510–1590 nm, using the onboard Gaussian light source, where the sampling frequency is set to 100 Hz. Figure 10 shows a sample EFPI measurement. Using the first two peaks in Figure 10, Equation (1) is used to provide an estimation of the cavity length. In this example, the cavity has an estimated length of 59 μm. As the pressure outside the cavity increases/decreases, the interference pattern can be seen to shift left and right. In the case of increasing external pressure, the cavity is shortened as a result of the diaphragm deflecting inward, which results in the spectrum shifting towards shorter wavelengths. Figure 11 shows a typical example of changing interference pattern as a result of increasing external pressure.

### 2.5. Sensor Testing Using a Thermogravimetric Analyser

**Figure 12:**
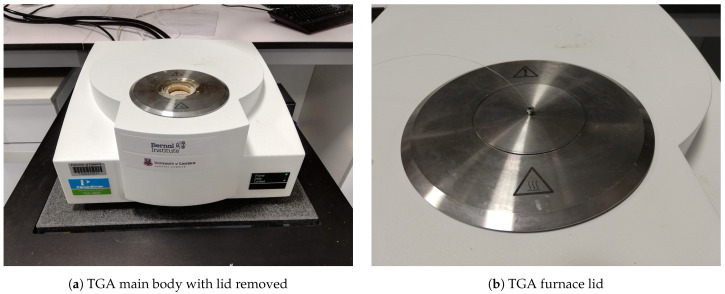
(**a**) TGA main body; (**b**) TGA furnace lid with a sensor SMF exiting.

In order to observe the temperature sensors’ thermal response in a relatively controlled environment, the sensors are heated in a thermogravimetric analyser (TGA) between an indicated 30–100 °C over a period of 14 min. The TGA used in this study is a Perkin Elmer (Waltham, MA, USA) TGA 4000. As the focus is solely on the qualitative behaviour of the temperature sensors, there are no sample materials placed in the TGA sample crucible during the tests. The temperature sensor is introduced into the TGA through the port located on the furnace lid, where it is lowered until it touches the sample crucible. The temperature sensor is subsequently retracted until it is approximately halfway between the inner face of the furnace lid and the sample crucible. Figure 12 shows the lid of the TGA where the sensor SMF may be seen exiting out of the furnace lid.

## 3. Results

**Figure 13:**
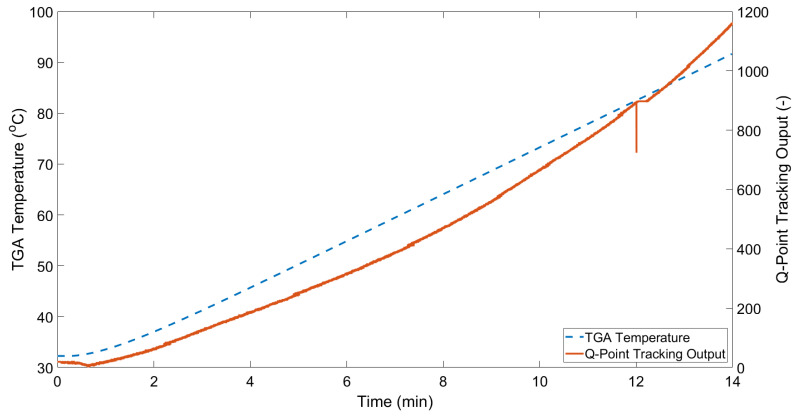
Change of intensity at 1541 nm in the reference all-glass UHRTS interference pattern.

**Figure 14:**
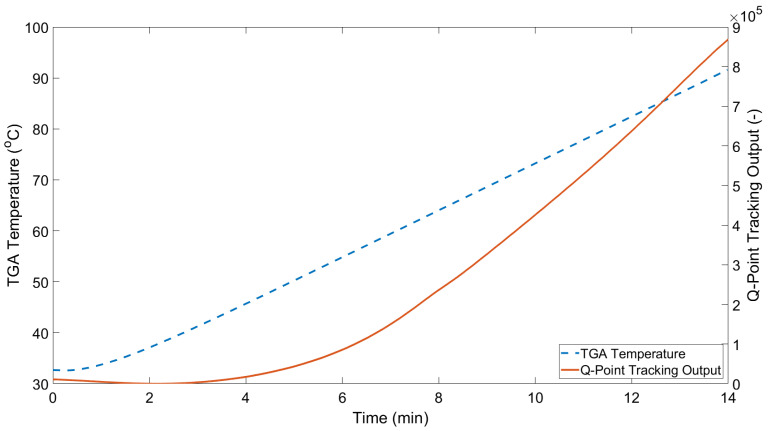
Change of intensity at 1541 nm in the PVA diaphragm based temperature sensor interference pattern.

**Figure 15:**
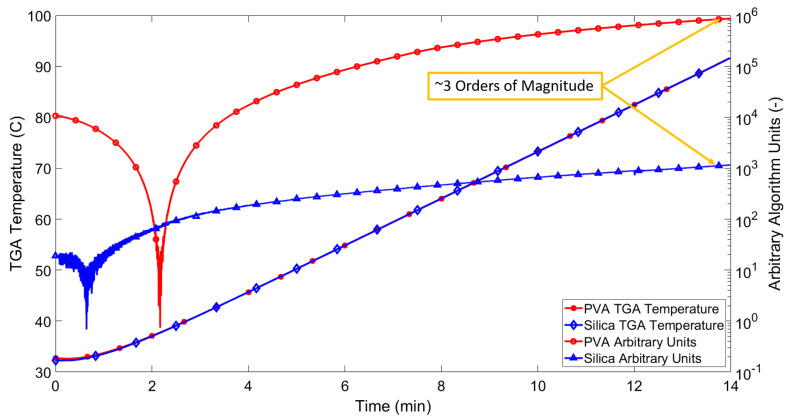
Overlay of TGA measurements and sensor responses.

As the TGA temperature increases, the fluid in the outer capillary undergoes volumetric expansion in line with its coefficient of thermal expansion. As the outer capillary is sealed, this leads to an increase of the oil pressure, thereby deflecting the OFPS diaphragm. In effect, the outer capillary and oil act as a transducer of temperature to pressure. The blue lines in Figure 13 and Figure 14 show the change of normalised intensity at an arbitrary wavelength based on tracking of the sensor’s Q-Point. The chosen Q-Point is based on a linear region of the baseline interference pattern, i.e., approximately halfway between a peak and a trough of the spectral response. The solid orange lines show the increasing sample temperature based on the TGA readout. Note that the readout temperature is slightly lower than that of the programmed temperature range; this is caused by the thermal inertia of the furnace itself. Thermal inertia is the delay between a command being sent to increase the furnace temperature and the heating element reaching the target temperature. Furthermore, as the cooling of the TGA is not controlled, the TGA temperature readout ceases at the point of maximum temperature (100 °C) indicated. As such, only the 14 min of comparable data recorded by the temperature sensor is of interest. Figure 13 shows an approximately linear response from 0–0.5 min for both the TGA readout and the all-glass UHRTS. As the temperature begins increasing in a relatively linear fashion for the TGA, the all-glass UHRTS appears to exhibit a low order polynomial response. Figure 14 again shows the relatively linear response from 0–0.5 min for the TGA temperature readout. Figure 15 shows an overlay of both the silica and PVA sensor measurements, along with their respective TGA readouts. Due to the significant difference in apparent sensitivities, both sensor responses are plotted on a semi-log scale. By plotting on a semi-log scale, the initial decrease in TGA temperature as seen in Figure 13 and Figure 14 is also made more apparent. However, the point at which the minimum TGA temperature is observed is delayed in the PVA sensor when compared to the TGA readout and the silica sensor response. One possible reason for the delay in the PVA sensor reaching the minimum is discussed in Section 4.2.

## 4. Discussion

### 4.1. Discussion on the Manufacturing Process

This section provides a brief discussion of the manufacturing process utilised in the production of the novel sensors. As noted in Section 1, while the all-glass construction of the reference OFPTS used to create a UHRTS is reasonably matured [15], it is not without drawbacks. The main manufacturing challenges for the all-glass based temperature sensors include: (a) the high failure rate of the all-glass construction at the SMF-capillary fusion point; and (b) diaphragm perforation during HF etching. While the all-glass sensors that are successfully produced give reliable and repeatable results, the authors are of the opinion that the process would benefit from the following changes: (a) mechanical strengthening of the fusion point between the stripped SMF and the capillary; (b) mechanical strengthening of the diaphragm; and (c) removal of the requirement for HF in the fabrication process.

Initial experiments with an optical fibre re-coater were undertaken to determine whether or not the exposed SMF and capillary fusion point could be accurately re-coated without coating the diaphragm. This may be a viable option if a suitable high-precision optical fibre re-coater, in which the optical fibre is not held at both ends under tension, becomes commercially available. While this solution would address the issue of sensor shearing at the SMF-capillary interface, it did not result in a stronger diaphragm, nor does it remove the risk of the diaphragm becoming perforated during etching in HF. A further issue with the conventional fibre optic re-coating approach is controlling the thickness of material that is deposited on the sensor diaphragm. While it would have been considered ideal to have some coating on the diaphragm for protection, the ultraviolet curable coating used in a commercial re-coater pooled at the end-face of the sensor and covered the diaphragm, thereby significantly reducing sensitivity.

As described, the use of PVA creates an artefact-free diaphragm once cured. The diaphragm is formed on the end of a diaphragm-less SMF-capillary assembly by introducing and extracting vertically the assembly into the PVA adhesive using the immersion rig in Figure 6b. The extraction rate being the parameter vital to controlling the coating thickness at the SMF-capillary junction and additionally controlling the diaphragm thickness. The results of Section 3 and the observations of Section 2.2 are now compared to establish the qualitative effect that the novel PVA diaphragm has on the performance of an EFPI based temperature sensor compared to the reference all-glass design.

### 4.2. Discussion on the Experimental Results

Referring to Figure 13, and as noted in Section 3, there is minimum net increase in TGA temperature for the initial minute of the TGA temperature programme cycle. A small decrease in temperature is also noted in the first several seconds of the TGA program cycle. The observed temperature decrease in the TGA chamber is caused by the introduction of nitrogen gas (N_2_) at the start of the TGA programme cycle to purge the TGA chamber of oxygen (O_2_) in order to help prevent a potential fire and/or explosion. It is reasonably assumed that the expansion of the nitrogen gas is what causes the observed initial temperature decrease. The TGA readout temperature begins increasing around 30 s into the program. However, a notable lag is seen in the response of the silica sensor, which shows no appreciable increase until approximately 1 min 30 s into the TGA program. The likely reason for this is that as the crucible temperature increases, there will be a period of time before the air temperature above the TGA crucible increases. Once the oil temperature begins to increase, the sensor response is seen to follow a low-order polynomial curve despite the TGA temperature ramping in a relatively linear fashion. A number of factors may be contributing to this polynomial response. The first of these relates to the construction of the sensor itself; as the sensor is heated i, t may be hypothesised that the silica components also undergo thermal expansion, which acts to increase the length/diameter of both the oil filled cavity and the inner cavity of the OFPS. Coupled with thermally induced mechanical expansion of the sensor structure, as the temperature increases, air in the sensor cavity will also expand and create an opposing force on the deflection of the diaphragm.

Most notable from observing Figure 14 is that there is a greater time lag between the ramping of the TGA temperature and that of the reference all-glass design. Initially, this observation may appear somewhat counter-intuitive given the lower specific heat capacity of PVA compared to silicon dioxide. The difference in the relative response times between the all-glass and PVA-based sensors may be explained by factors such as (a) the increased thermal mass of the PVA sensor (large deposit of PVA on sensor body) and (b) the compliance of the PVA, which will absorb some of the oils expansion in volume. Similar to the reference all-glass design, an initial drop in temperature is observed while testing the PVA based temperature sensor. The lowest temperature measurement for the glass sensor occurs at 45 s, while the lowest temperature measurement for the PVA sensor is recorded in the region of 2 min. Potential compressibility of the PVA encapsulating the sensor body when compared to the silica diaphragm may act to dampen the response; however, further investigation is necessary to determine whether or not this is the case. Figure 15 displays the temperature responses in a logarithmic format for ease of comparison, owing to the apparent higher sensitivity the PVA exhibits relative to the all-glass design. The PVA response approaches three orders of magnitude greater than the all-glass design at the end of the TGA programme (highlighted by yellow arrow in Figure 15). The PVA sensor is a potential solution to the criteria outlined in Section 1 as the PVA sensor is both easier to manufacture and provides some level of protection to the exposed silica components. The main reason for the apparent increased sensitivity, as hypothesised by the authors, is the extremely low Young’s Modulus of PVA (0.27 GPa) compared to silicon-dioxide (180 GPa). Referring to Equation (2), the diaphragm deflection is inversely proportional to the Young’s Modulus of the diaphragm material. In addition to the lower Young’s Modulus (E), for equally sized outer capillaries, the PVA based temperature sensor has a lower oil volume, due to the greater volume displaced by the PVA OFPS head; this in turn will produce a greater pressure increase in the sensors outer capillary for equivalent heat transfer.

### 4.3. Future Work

The work presented here regarding the use of PVA focused solely on using a single constant extraction rate to form the PVA diaphragm. Further work will explore the potential benefits of using a modulated extraction rate. Furthermore, as only a diaphragm created using the single constant extraction rate was tested, there likely exists some advantage to using a number of different extraction rates during the extraction of a single sensor body. Further improvements to the Q-Point tracking algorithm that determines cavity length are required in order to maximise the benefits of the potentially high-sensitivity response observed in the PVA sensor. A high-fidelity multi-physics model is currently being developed in Comsol Multiphysics (Stockholm, Sweden) version 5.6 and will be used in conjunction with the aforementioned improvements in manufacturing and signal processing to more fully understand the lag in the PVA sensor’s response. The multi-physics model will also be utilised to help guide the sensor’s design.

Furthermore, while the PVA diaphragm temperature sensor presented herein shows potentially significant temperature sensitivity; no detailed calibration has yet been undertaken; nonetheless, change in the cavity length per 1 K is estimated to be in the range of 0.42 μm. The next phase of research will focus on an accurate assessment of the temperature resolution. Previous research at the University of Limerick has shown that for the reference all-glass design, a theoretical resolution of 1.04 mK could be achieved. Considering the theoretical temperature resolution of the prior all-glass based UHRTS and the orders of magnitude higher sensitivity of the PVA-based sensor, it is reasonable to conjecture that the PVA-based sensor may prove to have a temperature resolution in the micro-Kelvin range (μK); however, rigorous empirical analysis, steady-state testing and further experimentation is needed before this can be fully determined.

## 5. Patents

The sensor manufacturing technique presented herein when used in conjunction with a hybrid sol-gel is currently patent pending.

## Figures and Tables

**Table 1:** Fusion Splicer Settings.

Parameter	Value	Unit
Prefusion	1	s
Prefusion Current	10	mA
Gap	50	μm
Overlap	10	μm
Fusion Time 1	0.03	s
Fusion Current 1	10.5	mA
Fusion Time 2	10	s
Fusion Current 2	11	mA
Fusion Time 3	1	s
Left MFD	9.8	μm
Right MFD	9.8	μm
Set Centre	255	-
AOA Current	0	mA
Early Prefuse	No	-

**Table 2:** Fusion Splicer Settings for Capillary Collapse.

Parameter	Value	Unit
Cleaning Current	10.0	mA
Cleaning Time	0.05	s
z-gap	3.00	μm
Autofeed	0.00	μm
Prefusion Current	10.0	mA
Prefusion Time	2.50	s
Fusion Current	10.0	mA
Fusion Time	10.0	s
Tensile Test	No	-

## Data Availability

Samples of the particular hybrid sol-gel noted are unavailable due to commercial interests. The PVA used is commercially available under the brand name it’s a stick up—pva wood glue.

## Abbreviations

The following abbreviations are used in this manuscript:

AOA	Arc-on Alignment
EFPI	Extrinsic Fabry-Perot Interferometer
FBG	Fibre-Bragg Grating
HF	Hydrofluoric Acid
IPA	Isopropyl Alcohol
MFD	Mode Field Diameter
MMF	Multimode Fibre
OFPS	Optical Fibre Pressure Sensor
OSA	Optical Spectrum Analyser
PVA	Polyvinyl Alcohol
SAE	Society of Automotive Engineers
SMF	Single Mode Fibre
TGA	Thermogravimetric Analyser
UHRTS	Ultra High Resolution Temperature Sensor
